# Data on the lipoprotein (a), coronary atherosclerotic burden and vulnerable plaque phenotype in angiographic obstructive coronary artery disease

**DOI:** 10.1016/j.dib.2016.04.017

**Published:** 2016-04-13

**Authors:** Giampaolo Niccoli, Diana Chin, Giancarla Scalone, Mario Panebianco, Sofia Abbolito, Nicola Cosentino, Francesca Jacoangeli, Hesham Refaat, Giovanna Gallo, Gerardo Salerno, Massimo Volpe, Filippo Crea, Luciano De Biase

**Affiliations:** a Cardiology Department, Catholic University of the Sacred Heart, Rome, Italy; b Cardiology Department, Department of Clinical and Molecular Medicine, 2nd School of Medicine, University of Rome “Sapienza”, S. Andrea Hospital, Rome, Italy; c Thorax Institute, Hospital Clinic, University of Barcelona, Spain; d Cardiology Department, Zagazig University, Zagazig, Egypt

**Keywords:** Acute coronary syndrome, Lipoprotein (a), Angiographic analysis, Optical coherence tomography

## Abstract

Lipoprotein Lp(a) represents an independent risk factor for coronary artery disease (CAD). However, its association with CAD burden and lipid rich plaques prone to rupture in patients with acute coronary syndrome (ACS) still remains unknown. These data aim to investigate the association among serum Lipoprotein(a) (Lpa) levels, coronary atherosclerotic burden and features of culprit plaque in patients with ACS and obstructive CAD. For his reason, a total of 500 ACS patients were enrolled for the angiographic cohort and 51 ACS patients were enrolled for the optical coherence tomography (OCT) cohort. Angiographic CAD severity was assessed by Sullivan score and by Bogaty score including stenosis score and extent index, whereas OCT plaque features were evaluated at the site of the minimal lumen area and along the culprit segment. In the angiographic cohort, Lp(a) was a weak independent predictor of Sullivan score (*p*<0.0001), stenosis score (*p*<0.0001) and extent index (*p*<0.0001). In the OCT cohort, patients with higher Lp(a) levels (>30 md/dl) compared to patients with lower Lp(a) levels (<30 md/dl) exhibited a higher prevalence of lipidic plaque at the site of the culprit stenosis (*P*=0.02), a wider lipid arc (*p*=0.003) and a higher prevalence of thin-cap fibroatheroma (*p*=0.004)

## **Specifications Table**

**Table:** 

Subject area	*Medical Clinical Research*
More specific subject area	*Coronary Intervention; Angiography; Intra-coronary Imaging; lipids*
Type of data	*Figure*
How data was acquired	*Blood sampling*
Data format	*Analyzed*
Experimental factors	*Venous blood samples were drawn and then centrifuged in appropriate tubes; aliquots of serum were stored at −80 °C until assayed.*
Experimental features	*Lipoprotein(a) was assessed with a test immuno-turbidimetric boosted with particles: human LPA agglutinates latex particles coated with antibodies to Lipoprotein(a); the precipitate is determined turbidimetrically at 552 nm.*
Data source location	*Policlinico Gemelli, Rome, Italy; Sant Andrea hospital, Rome, Italy*
Data accessibility	*The data is with this article*

**Value of the data**

• *These data prime prospective studies designed in order to address the effect of lipid lowering drugs on Lipoprotein(a) (Lpa) value.*
• *These data may prime future studies addressing the role of therapies targeting Lp(a) in the reduction of vulnerable plaque features of patients with coronary artery disease (CAD).*
• *These data may prime multicenter evaluation of the association between optical coherence tomography (OCT) features and Lp(a) values.*

## 1 Data

These data exhibit a positive correlation between Lp(a) levels and coronary atherosclerotic burden assessed by angiography in patients with acute coronary syndrome (ACS) (Sullivan Score *R*^2^=0.35, *p*<0.0001; Stenosis score *R*^2^=0.21, *p*<0.0001); Extent index (*R*^2^=0.24, *p*<0.0001).

**Fig. 1:**
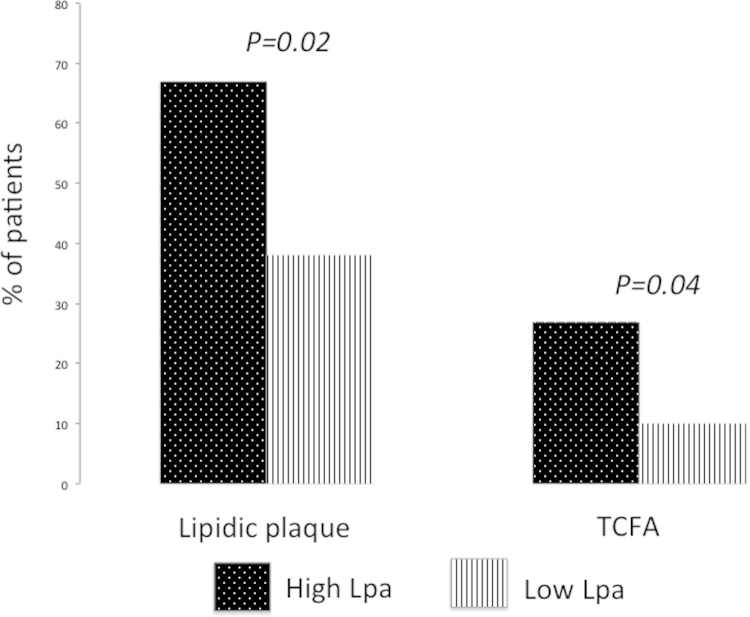
The prevalence of lipidic plaque and thin-cap fibroatheroma (TCFA) in the patients with high Lipoprotein(a) (Lpa) value vs. those with low Lipoprotein(a) value.

Moreover, patients with Lp(a) levels>30 md/dl compared to patients with Lp(a) levels<30 md/dl exhibited a higher prevalence of lipidic plaque at the site of the culprit stenosis (67% vs. 27%; *P*=0.02) (Fig. 1), a wider lipid arc (135±114 vs. 59±111; *P*=0.03) and a higher prevalence of thin-cap fibroatheroma (38% vs. 10%; *P*=0.04) (Fig. 1).

## 2 Experimental design, materials and methods

A total of 500 patients (370 men, average age 66±11) with ACS were prospectively enrolled for the angiographic evaluation of CAD severity with Sullivan score and by Bogaty score including stenosis score and extent index, whereas 51 ACS patients (29 males, average age 65±11) were retrospectively included for OCT examination.

In order to investigate the features of coronary atherosclerosis in patients with normal vs. abnormal Lp(a), angiographic and OCT cohort were divided in two groups: those with Lp(a) values<30 mg/dl and those with Lp(a)≥30 mg/dl.

*Statistical analysis*: A multiple linear regression was performed to assess independent predictors of the angiographic scores.

### 2.1 Angiographic analysis

Two expert angiographers, blinded to the laboratory values, evaluated all angiographic images to assess presence, severity and extent of CAD. Any disagreement between the two angiographers was resolved by consensus; when consensus could not be reached, a third experienced angiographer assessed and classified the coronary lesions.

The Bogaty׳s score assesses disease severity and extent. Severity of disease refers to the total number of ≥50% narrowings in all vessels of the angiogram. A maximum of three stenoses was permitted per coronary arterial segment. Extent of disease is obtained by dividing the extent score of the entire coronary arterial tree by the number of analyzed segments. A segment is scored 0 if it appeared angiographically normal, 1 if ≤10% of its length appeared abnormal, 2 if >10% up to 50% of its length was abnormal, and 3 if >50% of its length was abnormal. Since there were 15 segments considered, the extent index could vary from 0 to 3.

Stenosis score refers to the most severe stenosis observed in each main coronary artery branch. Grading is as follows: grade (1) 1–49% reduction in coronary lumen diameter; grade (2) stenosis with 50–74% reduction in lumen diameter; grade (3) stenosis with 75–99% reduction in lumen diameter; and grade (4) total coronary occlusions. The score is obtained by the sum of the results in each main coronary vessel (maximal value 32, if all eight main coronary artery branches have grade 4).

The extent score refers to the proportion of the coronary artery tree showing angiographically detectable atheroma. The observed proportion in each vessel is multiplied by a factor that varies according to the artery involved: left main stem, 5; left anterior descending coronary artery, 20; main diagonal branch, 10; first septal perforator, 5; left circumflex artery, 20; obtuse marginal and postero-lateral vessels, 10; right coronary artery, 20; and main posterior descending branch, 10. When the major lateral wall branch was a large obtuse marginal or intermediate vessel, this was given a factor of 20, and the left circumflex artery, a factor of 10. When a vessel was occluded and the distal bed was not fully visualized by collateral flow, the proportion of vessel not visualized was given the mean extent score of the remaining vessels. The scores for each vessel or branch were added to give a total score (maximal value=100); this total score represents an estimate of the percentage of the coronary luminal surface area involved by atheroma.

### 2.2 Frequency domain-optical coherence tomography (FD-OCT) analysis

FD-OCT analysis was divided into two parts: the analysis performed at minimal lumen area (MLA) site and the analysis conducted along the culprit vessel. FD-OCT analysis was performed at MLA site. OCT analysis was targeted on plaque characterization, presence of plaque rupture, fibrous cap thickness, presence of thin-cap fibro-atheroma, presence of intracoronary thrombus and minimal lumen area. Plaques assessed at the site of minimal lumen area were classified as lipidic, fibrous or calcified plaques. Lipid pool was defined as homogenous, diffusely bordered, signal-poor regions with overlying signal-rich bands. Fibrous plaques were defined as homogeneous high signal regions. Calcified plaques were defined as heterogeneous or signal poor regions with sharply delineated borders. Plaque rupture was defined as the presence of fibrous cap discontinuity leading to a communication between the inner necrotic core of the plaque and the lumen, with or without the presence of a flap. Thin-cap fibroatheroma was defined as a lipid-rich plaque with a fibrous cap thickness of ≤65 µm. Intra-coronary thrombus was defined as an irregular mass protruding into the lumen or adjacent to the luminal surface. Microchannels were defined as non-signal tubule-luminal structures without a connection to the vessel lumen, recognized on more than three consecutive cross-sectional OCT images.

### 2.3 Statistical analysis

Continuous variables between the two groups of subjects in the study population (Lp<30 mg/dl and Lp≥30 mg/dl) were compared with unpaired *t*-test or Mann–Whitney *U*-test. Categorical variables were compared using the chi square test or Fisher׳s exact test, as appropriate. Correlations between variables were performed using the Pearson test or the Spearman׳s rank test, as appropriate. A multiple linear regression was performed to assess independent predictors of the Bogaty and Sullivan score. In the univariate model, we included the overall clinical and angiographic variable considered. On the other hand, in the multiple regression analysis model, we included all variables showed in the univariate except drugs. In particular, in the linear regression, analyses with stepwise variable selection were applied to assess independent correlates of stenosis, Sullivan and Extend score. Covariates were all clinical and angiographic variables. In order to address skewed data, logarithmic transformation was applied directly from the software. Studies have shown that values greater than 30 mg/dL are considered abnormal. *P*<0.05 was always required for statistical significance.

